# Rejuvenation of chicory and lettuce plants following phase change in tissue culture

**DOI:** 10.1186/s12896-019-0557-z

**Published:** 2019-09-11

**Authors:** Anthony J. Conner, Helen Searle, Jeanne M. E. Jacobs

**Affiliations:** 1 Bio-Protection Research Centre, Lincoln University, P.O. Box 85084, Canterbury, 7647 New Zealand; 2 Forage Science, AgResearch, Private Bag 4749, Christchurch, 8140 New Zealand

**Keywords:** *Cichorium intybus*, Chicory, *Lactuca sativa*, Lettuce, Compositae, In vitro flowering, Rejuvenation, Plant tissue culture

## Abstract

**Background:**

A frequent problem associated with the tissue culture of Compositae species such as chicory (*Cichorium intybus* L.) and lettuce (*Lactuca sativa* L.) is the premature bolting to in vitro flowering of regenerated plants. Plants exhibiting such phase changes have poor survival and poor seed set upon transfer from tissue culture to greenhouse conditions. This can result in the loss of valuable plant lines following applications of cell and tissue culture for genetic manipulation.

**Results:**

This study demonstrates that chicory and lettuce plants exhibiting stable in vitro flowering can be rejuvenated by a further cycle of adventitious shoot regeneration from cauline leaves. The resulting rejuvenated plants exhibit substantially improved performance following transfer to greenhouse conditions, with increased frequency of plant survival, a doubling of the frequency of plants that flowered, and substantially increased seed production.

**Conclusion:**

As soon as in vitro flowering is observed in unique highly-valued chicory and lettuce lines, a further cycle of adventitious shoot regeneration from cauline leaves should be implemented to induce rejuvenation. This re-establishes a juvenile phase accompanied by in vitro rosette formation, resulting in substantially improved survival, flowering and seed set in a greenhouse, thereby ensuring the recovery of future generations from lines genetically manipulated in cell and tissue culture.

## Background

Cell and tissue culture can be used in a wide range of applications for the genetic improvement of plants [1]. Efficient regeneration of shoots from cell cultures is important for applications such as embryo rescue following wide hybridisation, generation of somaclonal variation, somatic cell selection, somatic hybridisation, transformation with cloned genes, and genome editing. The effective transfer of the plants with putative genetic improvements from tissue culture conditions to the greenhouse is essential [2]. In a crop improvement context, the production of healthy greenhouse plants with good seed set subsequent to tissue culture is critical for integration of somatic approaches into genetic improvement programmes.

An inherent problem associated with the growth of chicory (*Cichorium intybus* L.) and lettuce (*Lactuca sativa* L.) plants in tissue culture involves the spontaneous premature in vitro bolting in some regenerated plants [3–6]. Once this phase change occurs in tissue culture it becomes a very stable epigenetic change that is maintained upon further micro-propagation of the plants. Anecdotal observations suggest that such chicory and lettuce plants exhibit poor growth and develop into plants with a spindly appearance that immediately flower upon transfer out of tissue culture to greenhouse environments. Furthermore, these plants have insufficient vegetative resources to sustain normal seed development. The occurrence of such in vitro phase changes during the applications of cell and tissue culture for genetic manipulation of chicory and lettuce (e.g. [7–12]) can result in the loss of otherwise valuable plant lines, thereby impacting upon the adoption of somatic approaches for genetic improvement.

This study validates the anecdotal observations on performance of chicory and lettuce plants exhibiting phase changes to flowering during in vitro culture. More importantly, it establishes the rejuvenation of chicory and lettuce plants following a further cycle of adventitious shoot regeneration from in vitro plants in the adult phase. The resulting rejuvenated plants exhibit substantially improved performance following transfer to greenhouse conditions by re-establishing a normal plant development cycle toward flowering and seed set.

## Results

**Fig. 1:**
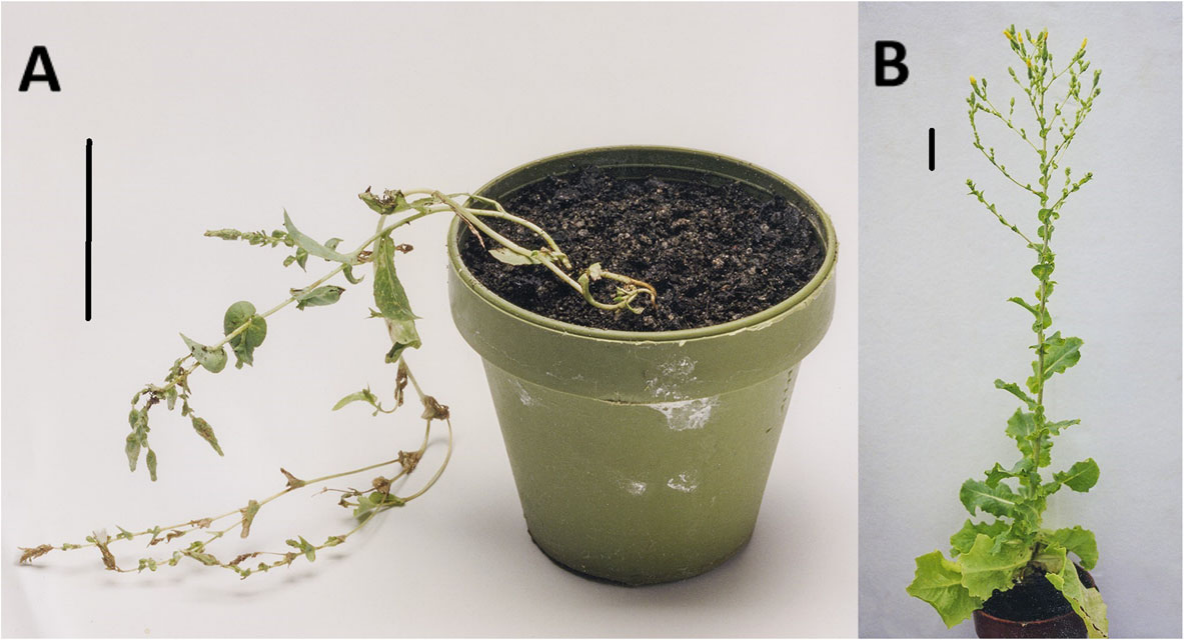
The appearance of lettuce plants (cv. ‘Cobham Green’) 12 weeks after transfer from in vitro culture to a greenhouse. Vertical scale bars represent 5 cm. **a** A plant derived from a shoot exhibiting premature bolting while being propagated in vitro. **b** A plant derived from a shoot exhibiting rosette development while being propagated in vitro

During the routine tissue culture of chicory and lettuce, spontaneous premature in vitro bolting was observed in some regenerated plants without rosette development. These in vitro adult-phase plants exhibited poor growth upon the transfer to greenhouse environments and immediately flowered (e.g. Figure 1a). In contrast, plants with in vitro rosette development established a normal growth cycle upon transfer out of tissue culture (e.g. Figure 1b). Experiments were therefore designed to validate these observations on performance of chicory (cultivar ‘Grasslands Puna’) and lettuce (cultivar ‘Cobham Green’) plants regenerated from tissue culture.

For both chicory and lettuce, a population of 50 plants were micropropagated from the nodal stem segments of plants that had undergone phase change to in vitro flowering and were subsequently transplanted to soil and transferred to greenhouse conditions (hereinafter referred to as adult-phase plants). At the same time, 50 plants derived from adventitious shoots regenerated from the cauline leaves (leaves on the flowering stems) of plants flowering in vitro were also transplanted to soil and transferred to greenhouse conditions for both chicory and lettuce (hereinafter referred to as rejuvenated plants). For both the adult-phase plants and the rejuvenated plants, no more than four individuals were derived from each germinated seed. The survival, developmental phase, flowering and/or seed production were recorded for these two sets of plants in the greenhouse.

**Fig. 2:**
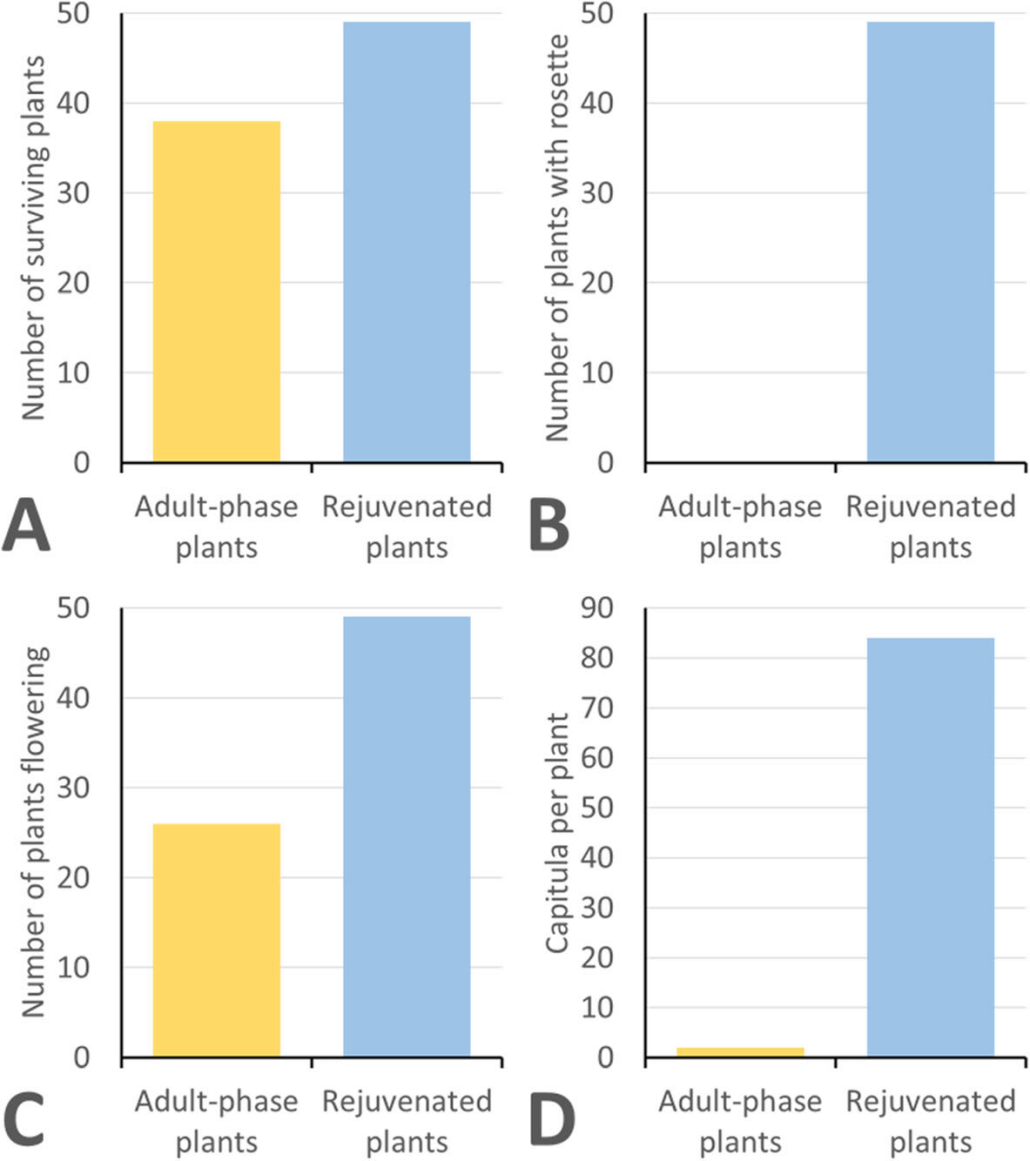
Performance of chicory plants cv. ‘Grasslands Puna’ upon transfer from tissue culture to a greenhouse. A total of 50 plants were transferred for both adult-phase plants and rejuvenated plants. Data on the individual 50 plants can be found in Additional file 1. **a** The number of plants surviving following transfer to the greenhouse (χ^2^ = 8.84, 1 df, *P* < 0.005). **b** The number of plants developing a rosette following transfer to the greenhouse (χ^2^ = 92.20, 1 df, *P* < 0.001). **c** The number of plants that flowered following transfer to the greenhouse (χ^2^ = 25.81, 1 df, *P* < 0.001). **d** The median number of capitula per plant that flowered following transfer to the greenhouse (*U* = 1273, n_1_ = 49, n_2_ = 26, *P* < 0.00001)

In chicory, none of the adult-phase plants developed a rosette phase in tissue culture, whereas all the rejuvenated plants developed rosettes in tissue culture. Upon transfer to soil in a greenhouse environment the adult-phase plants exhibited poor growth, with a spindly appearance and stems that failed to support themselves in an upright position (similar for that illustrated by lettuce in Fig. 1a). The chicory plants trapped in a stable adult-phase exhibited 76% survival upon transfer to the greenhouse, significantly less than the 98% survival for the rejuvenated plants (Fig. 2a). None of the adult-phase plants formed rosettes in the greenhouse, whereas 98% of the rejuvenated plants developed rosettes (Fig. 2b). For a few of the rejuvenated chicory plants (10%), rosette development only partially occurred. Only 52% of the adult-phase plants flowered, which was substantially less than the 98% for the rejuvenated plants (Fig. 2c). The adult-phase chicory plants that flowered exhibited poor reproductive success compared with the rejuvenated chicory plants (Fig. 2d). The median number of capitula (composite flower-heads) per adult-phase plant that flowered was only two (range of 0 to 8 capitula per plant), a highly significant lower value than the 84 capitula per plant for the rejuvenated plants (range of 7 to 141 capitula per plant). For the rejuvenated chicory plants, those with partial rosette development produced substantially less capitula (median = 21) than the plants with full rosette development (median = 89), with a highly significant difference in the distribution between the two plant populations as determined by a two-tailed Mann-Whitney *U*-test (*U* = 220, n_1_ = 44, n_2_ = 5, *P* = 0.0003).

**Fig. 3:**
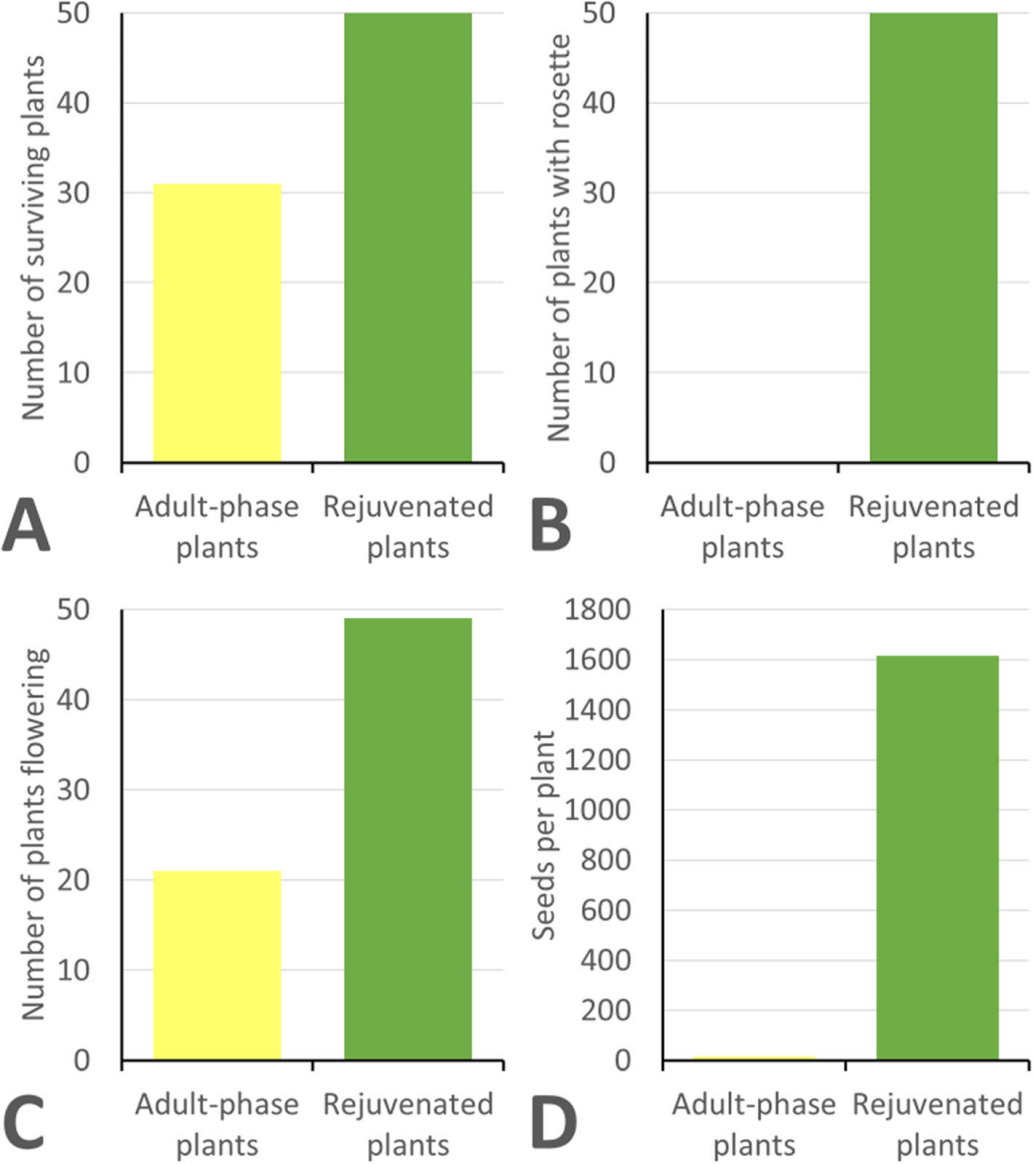
Performance of lettuce plants cv. ‘Cobham Green’ upon transfer from tissue culture to a greenhouse. A total of 50 plants were transferred for both adult-phase plants and rejuvenated plants. Data on the individual 50 plants can be found in Additional file 2. **a** The number of plants surviving following transfer to the greenhouse (χ^2^ = 21.05, 1 df, *P* < 0.001). **b** The number of plants developing a rosette following transfer to the greenhouse (χ^2^ = 96.04, 1 df, *P* < 0.001). **c** The number of plants that flowered following transfer to the greenhouse (χ^2^ = 34.71, 1 df, *P* < 0.001). **d** The median number of capitula per plant that flowered following transfer to the greenhouse (*U* = 1029, n_1_ = 49, n_2_ = 21, *P* < 0.00001)

Similar to chicory, none of the adult-phase lettuce plants developed a rosette phase in tissue culture, whereas all the rejuvenated lettuce plants developed rosettes in tissue culture. Upon transfer to soil in a greenhouse environment the adult-phase lettuce plants exhibited very poor growth, with a similar appearance and form as illustrated in Fig. 1a. All the rejuvenated lettuce plants survived transfer to the greenhouse, significantly more than the 62% for the adult-phase plants (Fig. 3a). None of the adult-phase plants formed rosettes in the greenhouse, whereas all the rejuvenated plants developed rosettes (Fig. 3b). However, the rosette formation was only partially developed in 36% of the rejuvenated lettuce plants. Flowering was observed in 98% of the rejuvenated lettuce plants, substantially more than the 42% for the adult-phase plants (Fig. 3c). For the key trait of seed production, the adult-phase lettuce plants performed poorly compared with the rejuvenated lettuce plants (Fig. 3d). The median number of seeds per adult-phase plant that flowered was only five (range of 0 to 21 seeds per plant), a highly significant lower number than the 1616 seeds per plant for the rejuvenated plants (range of 92 to 3356 seeds per plant). For the rejuvenated lettuce plants, those with full rosette development produced substantially more seeds (median = 2147) than the plants with partial rosette development (median = 262), with a highly significant difference in the distribution between the two plant populations as determined by a two-tailed Mann-Whitney *U*-test (*U* = 541, n_1_ = 32, n_2_ = 17, *P* < 0.00001).

## Discussion

Plant biotechnology offers a wide range of approaches for the genetic improvement of crops. For chicory and lettuce this includes applications of DNA markers in breeding programmes (e.g. [13]), as well as the use of cell and tissue culture for genetic improvement. These latter applications include embryo rescue for the recovery of interspecific hybrids [10], somaclonal variation [7], somatic cell selection [9], somatic fusion of protoplasts [8, 10], plant transformation [11], and gene editing [12].

An inherent problem associated with the growth of chicory and lettuce plants in tissue culture is the spontaneous premature in vitro flowering of regenerated plants. Once this phase change occurs in tissue culture it becomes a very stable epigenetic change that is maintained upon further micro-propagation of the plants. This study has exemplified earlier anecdotal observations on the difficulties associated with the recovery of useful plants following such in vitro phase changes [3]. In both chicory and lettuce these plants have poor survival upon transfer out of tissue culture to greenhouse environments (Figs. 2a and 3a). Plants that do survive the transfer to greenhouse conditions, exhibit poor growth and develop into plants with a spindly appearance that immediately flower (Fig. 1a). Such plants fail to form a normal rosette (Figs. 2b and 3b) and have insufficient vegetative resources to sustain normal seed development. This results in plants with very poor reproductive potential with respect to flowering and seed set (Figs. 2c, d, 3c and d). When cell and tissue culture is used for genetic improvement of chicory and lettuce, a consequence of spontaneous premature in vitro bolting among plants regenerated is the potential loss of otherwise valuable plant lines.

A key result from the present study in chicory and lettuce is a simple approach to induce reversion of the phase change associated with in vitro flowering. Phase change from a juvenile to an adult state has long been recognised as a stable physiological change in plants [14]. The stability of phase change is often attributed to epigenetic events associated with DNA methylation and histone modifications such as methylation and acetylation [15]. Phase change is regulated by the expression of highly conserved microRNAs, which act by repressing the expression of SBP/SPL transcription factors that further regulate a wide variety of genes associated with shoot development [16, 17]. Adult phase phenotypes undergo reversion to juvenile phase with a reset of plant development following sexual reproduction involving a cycle of meiosis, syngamy and zygotic embryogenesis [14]. Similarly, stable epigenetic changes arising in tissue culture are often lost upon plant regeneration [18].

When adult phase changes occur during the recovery of valuable genetic variants in chicory and lettuce tissue culture, rejuvenation can be induced by another cycle of adventitious shoot regeneration from the cauline leaves of the adult plants. This additional cycle of adventitious shoot regeneration resets the normal plant growth cycle, resulting in the development of a rosette growth form, thereby allowing plants to follow a normal flowering cycle and seed production in the greenhouse. The resulting rejuvenated plants exhibit superior performance following transfer to greenhouse conditions, with substantially higher survival rates and reproductive potential with respect to flowering and seed set (Figs. 2c, d, 3c and d).

Only very rare seeds developed in some capitula from a few of the chicory plants. Consequently, flowering was only assessed in chicory (Fig. 2c and d), whereas flowering and seed set were recorded in lettuce (Fig. 3c and d). The poor seed set in chicory was expected given the self-incompatible nature of chicory reproduction [19] and the fact that some plants were cloned via micropropagation. Furthermore, seed production in chicory is highly dependent on insects for cross pollination [20], which were generally absent in the greenhouse.

All of the rejuvenated chicory and lettuce plants exhibited rosette development in vitro. Upon transfer and further growth in the greenhouse the majority of plants showed full rosette development, although 10% of the chicory plants and 36% of the lettuce plants developed only partial rosettes. It is unclear whether these plants failed to fully revert to a juvenile phase, or whether they underwent a rapid phase change to flowering following full reversion. Relative to plants with partial rosettes, those with full rosette development produced over four times the number of capitula for chicory and eight times the number of seeds in lettuce.

## Conclusions

When in vitro flowering is observed in chicory and lettuce plants during the application of somatic approaches for genetic improvement, there is a major risk that highly-valued independent genetic events are lost upon transfer of the plants to greenhouse conditions. To circumvent this problem, as soon as in vitro flowering is observed in unique highly-valued plant lines, a further cycle of adventitious shoot regeneration from cauline leaves should be immediately implemented to induce rejuvenation. This will re-establish a juvenile phase accompanied by in vitro rosette formation, resulting in these plant lines exhibiting substantially improved survival and performance following transfer from tissue culture to a greenhouse environment. This rejuvenation establishes a normal developmental cycle of the plants, resulting in the desired flowering and/or seed set in a greenhouse, thereby ensuring the recovery of future generations from lines genetically manipulated in cell and tissue culture.

## Methods

Seeds from the chicory (*Cichorium intybus* L.) cultivar ‘Grasslands Puna’ (Wrightson Seeds, Canterbury, New Zealand) and lettuce (*Lactuca sativa* L.) cultivar ‘Cobham Green’ (J.W. Boyce Seedsmen, Cambridgeshire, United Kingdom) were surface-sterilised with 70% ethanol for 30 s, transferred to a solution of 1% NaClO with 2 drops of Tween 20 detergent per litre for 30 min, then rinsed 2–3 times with sterile distilled water. Seeds were surface-sterilised and germinated on medium containing SH (Schenk & Hilderbrandt) salts and vitamins [21], supplemented with 30 g l^− 1^ sucrose and 7 g l^− 1^ of bacteriological agar (Germantown, New Zealand).

Following germination plants were micropropagated on the same medium for 2–3 cycles and those bolting to adult-phase in vitro were selected and micropropagated for a further 2–3 cycles. Cauline leaves (leaves on the flowering stems) of in vitro plants were excised and cut in half longitudinally, and the resulting leaf segments were placed on a regeneration medium consisting of the SH medium used for seed germination, supplemented with 0.1 mg l^− 1^ indole acetic acid, 0.5 mg l^− 1^ kinetin and 0.05 mg l^− 1^ zeatin [22, 23]. After 20 days, the explants were transferred to fresh regeneration medium of the same composition for a further 20 days. The regenerated shoots were transferred to SH medium without growth regulators for root initiation, resulting in a population of plants derived from adventitious shoots. A second population of plants was derived by micropropagation on the SH medium without growth regulators using single node cuttings from the stems of plants that had bolted to adult-phase in vitro.

The pH of all media was adjusted to 5.8 prior to addition of agar. Except for zeatin, which was filter sterilized and added after autoclaving, all the components were sterilized by autoclaving at 121 °C and 103 kPa for 15 min. Molten media in 50 ml aliquots were then dispensed into ethylene oxide pre-sterilized plastic pottles of size 85 mm diameter × 35 mm height. All cultures were incubated at 23–24 °C with 16 h photoperiod supplied by cool-white fluorescent lamps (70–85 μmol m^− 2^ s^− 1^).

Plants with roots about 1 cm long were transferred to greenhouse conditions about 12–15 days following their last subculture. The gelled medium was washed from their roots in tepid water, and they were planted into plastic 6-plug cavity trays (Flight Plastics, Wellington, New Zealand) with individual cells of 4.5 cm × 4.5 cm × 5.5 cm. The soil mix consisted of composted pine bark/sand potting mix supplemented with a slow release fertiliser [24]. The plants were thoroughly watered and placed in a humidity tent with > 85% relative humidity, maintained by an air humidifier (Defensor, model 505-S) in a greenhouse with heating < 18 °C and ventilation > 22 °C and natural day light for summer months at Lincoln in New Zealand (latitude 43^o^38’ S). After 4 days, the plants were removed from the humidity tent and placed in the shade under the greenhouse benches. After a further week plants were transferred to the top of the benches to further acclimatise, then transplanted into the same potting mix in either 5-l plastic pots (lettuce) or 10-l plastic pots (chicory). Plants were watered as required and grown until flowering and seed set.

Survival, growth and development were monitored in two populations of plants for both chicory and lettuce. The plants derived from adventitious shoots regenerated from the cauline leaves were compared to those micropropagated from single nodes of adult-phase plants. The number of capitula (flower heads) was counted on each chicory plant after 5 months in the greenhouse. Seeds were harvested from mature lettuce plants and cleaned manually, and the total weight of seed from each plant was recorded. The number of lettuce seeds was calculated based on the 1000 seed weight being 1.246 g.

Individual plants were classified as positive or negative for survival, rosette formation and flowering for both the adult-phase and rejuvenated populations of plants. Chi-square tests of independence (2 × 2), using Yates’ correction for continuity were then used to test the null hypothesis of independence for each of the three traits. The significance of differences between the number of capitula or seeds per plants were assessed by a two-tailed Mann-Whitney *U*-test since the distribution of data did not fulfil the usual assumptions for standard parametric statistical analyses.

## Supplementary information

**Additional file 1.** Data from individual chicory ‘Grasslands Puna’ plants.

**Additional file 2.** Data from individual lettuce ‘Cobham Green’ plants.

## Data Availability

All data generated or analysed during this study are included in this published article (and its supplementary information files).
