# Supplementary material for: Rejuvenation of chicory and lettuce plants following phase change in tissue culture
Source: BMC Biotechnol. 2019 Sep 11;19:65. doi: 10.1186/s12896-019-0557-z (PMC6737603; doi:10.1186/s12896-019-0557-z)
Supplement: Supplementary file 1 — Additional file 1. Data from individual chicory ‘Grasslands Puna’ plants. [file 12896_2019_557_MOESM1_ESM.pdf]

**Additional file 1: Data from individual chicory 'Grasslands Puna' plants**

**Adult-phase *in vitro* plants upon transfer to a greenhouse**

| Plant # | Survive transfer to soil | Rosette phase | Survive to flower | Number of capitula |
|---|---|---|---|---|
| 1 | yes | no | yes | 1 |
| 2 | no | no | no | |
| 3 | yes | no | yes | 0 |
| 4 | yes | no | yes | 0 |
| 5 | yes | no | yes | 2 |
| 6 | yes | no | yes | 0 |
| 7 | yes | no | no | |
| 8 | yes | no | yes | 3 |
| 9 | yes | no | no | |
| 10 | no | no | no | |
| 11 | yes | no | yes | 2 |
| 12 | yes | no | yes | 1 |
| 13 | no | no | no | |
| 14 | yes | no | no | |
| 15 | yes | no | yes | 5 |
| 16 | no | no | no | |
| 17 | yes | no | yes | 2 |
| 18 | yes | no | no | |
| 19 | yes | no | yes | 0 |
| 20 | no | no | no | |
| 21 | yes | no | no | |
| 22 | yes | no | no | |
| 23 | yes | no | yes | 3 |
| 24 | no | no | no | |
| 25 | yes | no | yes | 2 |
| 26 | yes | no | yes | 0 |
| 27 | yes | no | no | |
| 28 | yes | no | yes | 2 |
| 29 | no | no | no | |
| 30 | yes | no | yes | 3 |
| 31 | yes | no | yes | 2 |
| 32 | yes | no | yes | 0 |
| 33 | no | no | no | |
| 34 | yes | no | yes | 2 |
| 35 | no | no | no | |
| 36 | yes | no | no | |
| 37 | yes | no | yes | 3 |
| 38 | yes | no | no | |
| 39 | yes | no | yes | 0 |
| 40 | yes | no | yes | 3 |
| 41 | no | no | no | |
| 42 | yes | no | yes | 0 |
| 43 | yes | no | no | |
| 44 | no | no | no | |

| | | | | |
|---|---|---|---|---|
| 45 | yes | no | yes | 8 |
| 46 | yes | no | no | |
| 47 | yes | no | yes | 1 |
| 48 | yes | no | yes | 0 |
| 49 | no | no | no | |
| 50 | yes | no | no | |

**Rejuvenated *in vitro* plants upon transfer to a greenhouse**

| Plant # | Survive transfer to soil | Rosette phase | Survive to flower | Number of capitula |
|---|---|---|---|---|
| 1 | yes | yes | yes | 67 |
| 2 | yes | yes | yes | 54 |
| 3 | yes | yes | yes | 78 |
| 4 | yes | yes | yes | 103 |
| 5 | yes | yes | yes | 48 |
| 6 | yes | yes | yes | 91 |
| 7 | yes | yes | yes | 88 |
| 8 | yes | partial | yes | 16 |
| 9 | yes | yes | yes | 57 |
| 10 | yes | yes | yes | 117 |
| 11 | yes | yes | yes | 93 |
| 12 | yes | yes | yes | 82 |
| 13 | yes | yes | yes | 121 |
| 14 | yes | yes | yes | 95 |
| 15 | yes | yes | yes | 81 |
| 16 | yes | yes | yes | 105 |
| 17 | yes | yes | yes | 133 |
| 18 | yes | yes | yes | 113 |
| 19 | yes | yes | yes | 107 |
| 20 | yes | yes | yes | 84 |
| 21 | yes | yes | yes | 77 |
| 22 | yes | yes | yes | 52 |
| 23 | yes | partial | yes | 7 |
| 24 | yes | yes | yes | 69 |
| 25 | yes | yes | yes | 126 |
| 26 | yes | yes | yes | 141 |
| 27 | no | no | no | 0 |
| 28 | yes | yes | yes | 64 |
| 29 | yes | yes | yes | 104 |
| 30 | yes | yes | yes | 118 |
| 31 | yes | partial | yes | 43 |
| 32 | yes | yes | yes | 51 |
| 33 | yes | yes | yes | 64 |
| 34 | yes | yes | yes | 86 |
| 35 | yes | yes | yes | 122 |
| 36 | yes | yes | yes | 53 |
| 37 | yes | yes | yes | 72 |
| 38 | yes | yes | yes | 126 |

| | | | | |
|---|---|---|---|---|
| 39 | yes | yes | yes | 61 |
| 40 | yes | partial | yes | 18 |
| 41 | yes | yes | yes | 93 |
| 42 | yes | yes | yes | 137 |
| 43 | yes | yes | yes | 109 |
| 44 | yes | yes | yes | 74 |
| 45 | yes | yes | yes | 98 |
| 46 | yes | yes | yes | 118 |
| 47 | yes | yes | yes | 65 |
| 48 | yes | partial | yes | 21 |
| 49 | yes | yes | yes | 77 |
| 50 | yes | yes | yes | 103 |
