# Supplementary material for: Rejuvenation of chicory and lettuce plants following phase change in tissue culture
Source: BMC Biotechnol. 2019 Sep 11;19:65. doi: 10.1186/s12896-019-0557-z (PMC6737603; doi:10.1186/s12896-019-0557-z)
Supplement: Supplementary file 2 — Additional file 2. Data from individual lettuce ‘Cobham Green’ plants. [file 12896_2019_557_MOESM2_ESM.pdf]

Additional file 2: Data from individual lettuce 'Cobham Green' plants

Adult-phase *in vitro* plants upon transfer to a greenhouse

Plant #	Survive transfer to soil	Rosette phase	Survive to flower	Number of seeds
1	yes	no	yes	4
2	yes	no	yes	8
3	no	no	no	
4	yes	no	yes	2
5	no	no	no	
6	yes	no	yes	5
7	no	no	no	
8	no	no	no	
9	yes	no	no	
10	yes	no	yes	2
11	no	no	no	
12	yes	no	yes	7
13	no	no	no	
14	yes	no	yes	21
15	yes	no	yes	0
16	no	no	no	
17	yes	no	yes	6
18	no	no	no	
19	yes	no	yes	8
20	no	no	no	
21	yes	no	no	
22	no	no	no	
23	yes	no	yes	5
24	yes	no	no	
25	yes	no	yes	14
26	no	no	no	
27	yes	no	no	
28	yes	no	yes	3
29	no	no	no	
30	yes	no	yes	9
31	yes	no	no	
32	yes	no	no	
33	yes	no	yes	12
34	no	no	no	
35	no	no	no	
36	yes	no	yes	2
37	yes	no	no	
38	yes	no	yes	4
39	no	no	no	
40	yes	no	yes	0
41	yes	no	no	
42	no	no	no	

43	no	no	no	
44	yes	no	yes	2
45	yes	no	no	
46	yes	no	no	
47	yes	no	yes	0
48	no	no	no	
49	no	no	no	
50	yes	no	yes	6

Rejuvenated *in vitro* plants upon transfer to a greenhouse

Plant #	Survive transfer to soil	Rosette phase	Survive to flower	Weight of seeds (g)	Number of seeds[a]
1	yes	yes	yes	1.073	861
2	yes	yes	yes	2.964	2379
3	yes	partial	yes	0.483	388
4	yes	yes	yes	4.182	3356
5	yes	partial	yes	0.133	107
6	yes	yes	yes	3.961	3179
7	yes	partial	yes	0.692	555
8	yes	yes	yes	1.765	1417
9	yes	yes	yes	0.738	592
10	yes	yes	yes	2.693	2161
11	yes	partial	yes	0.602	483
12	yes	partial	yes	0.497	399
13	yes	yes	yes	3.451	2770
14	yes	yes	yes	0.973	781
15	yes	yes	yes	2.057	1651
16	yes	partial	yes	0.115	92
17	yes	yes	yes	3.822	3067
18	yes	partial	yes	0.327	262
19	yes	yes	yes	3.473	2787
20	yes	yes	yes	1.836	1474
21	yes	partial	no	0	0
22	yes	yes	yes	2.385	1914
23	yes	partial	yes	1.139	914
24	yes	yes	yes	3.827	3071
25	yes	yes	yes	2.019	1620
26	yes	yes	yes	3.992	3204
27	yes	partial	yes	0.144	116
28	yes	yes	yes	2.658	2133
29	yes	partial	yes	0.291	234
30	yes	partial	yes	0.382	307
31	yes	yes	yes	4.012	3220
32	yes	yes	yes	2.368	1900
33	yes	partial	yes	0.534	429

34	yes	yes	yes	3.217	2582
35	yes	yes	yes	3.442	2762
36	yes	yes	yes	2.139	1717
37	yes	yes	yes	2.536	2035
38	yes	partial	yes	0.169	136
39	yes	partial	yes	0.281	226
40	yes	yes	yes	2.014	1616
41	yes	partial	yes	0.257	206
42	yes	yes	yes	3.886	3119
43	yes	yes	yes	3.013	2418
44	yes	yes	yes	1.862	1494
45	yes	yes	yes	2.715	2179
46	yes	partial	yes	0.377	303
47	yes	yes	yes	2.794	2242
48	yes	partial	yes	0.128	103
49	yes	yes	yes	2.318	1860
50	yes	yes	yes	1.736	1393

[a]based on 1000 seed weight = 1.246 g.
